# Emergence and Rapid Diagnosis of *Talaromyces marneffei* Infections in Renal Transplant Recipients by Next-Generation Sequencing

**DOI:** 10.1007/s11046-024-00898-3

**Published:** 2024-10-10

**Authors:** Fanfan Xing, Chaowen Deng, Shan Zou, Chi-Ching Tsang, Simon K. F. Lo, Susanna K. P. Lau, Patrick C. Y. Woo

**Affiliations:** 1 Department of Infectious Diseases and Microbiology, The University of Hong Kong – Shenzhen Hospital, Shenzhen, Guangdong China; 2 Department of Adult Intensive Care, The University of Hong Kong – Shenzhen Hospital, Shenzhen, Guangdong China; 3 School of Medical and Health Sciences, Tung Wah College, Homantin, Hong Kong China; 4 Department of Microbiology, School of Clinical Medicine, Li Ka Shing Faculty of Medicine, The University of Hong Kong, Pokfulam, Hong Kong, China; 5 Doctoral Program in Translational Medicine and Department of Life Sciences, National Chung Hsing University, Taichung 402, Taiwan; 6 The iEGG and Animal Biotechnology Research Center, National Chung Hsing University, Taichung 402, Taiwan

**Keywords:** *Talaromyces marneffei*, Renal transplant, Next-generation sequencing, Rapid diagnosis

## Abstract

In the last few years, next-generation sequencing (NGS) has emerged as a technology for laboratory diagnosis of many culture-negative infections and slow-growing microorganisms. In this study, we describe the use of metagenomic NGS (mNGS) for rapid diagnosis of *T. marneffei* infection in a 37-year-old renal transplant recipient who presented with chronic pneumonia syndrome. Bronchoalveolar lavage for mNGS was positive for *T. marneffei* sequence reads. Prolonged incubation of the bronchoalveolar lavage revealed *T. marneffei* colonies after 6 days of incubation. Analysis of 23 cases of *T. marneffei* infections in renal transplant recipients from the literature revealed that the number of cases ranged from 1 to 4 cases per five years from 1990 to 2020; but increased rapidly to 9 cases from 2021 to 2023, with 7 of them diagnosed by NGS. Twenty of the 23 cases were from *T. marneffei*-endemic areas [southern part of mainland China (*n* = 9); Hong Kong (*n* = 4); northeastern India (*n* = 2); Indonesia (*n* = 1) and Taiwan (*n* = 4)]. For the 3 patients from non-*T. marneffei*-endemic areas [United Kingdom (*n* = 2) and Australia (*n* = 1)], they had travel histories to China and Vietnam respectively. The time taken for diagnosis by mNGS [median 1 (range 1 to 2) day] was significantly shorter than that for fungal culture [median 6 (range 3 to 15) days] (*P* = 0.002). mNGS is useful for picking up more cases of *T. marneffei* infections in renal transplant recipients as well as providing a rapid diagnosis. Talaromycosis is an emerging fungal infection in renal transplant recipients.

## Introduction

*Talaromyces* (*Penicillium*) *marneffei* is the most important pathogenic thermally dimorphic fungus causing systemic mycosis in Southeast Asia [1–3]. *T. marneffei* infection is endemic in tropical regions, especially Thailand, Vietnam, northeastern India, Southern China, Hong Kong, Taiwan, Laos, Malaysia, Myanmar, Cambodia and Laos [4]. Bamboo rats (*Rhizomys* spp. and *Cannomys* spp.) and soil from their burrows are considered to be important enzootic and environmental reservoirs of *T. marneffei*, respectively [5–7]. Historically, *T. marneffei* infection in human has been considered to be almost exclusively associated with acquired immunodeficiency syndrome (AIDS) caused by human immunodeficiency virus (HIV) infection [4, 8]. In some regions such as Hong Kong and southern China, *T. marneffei* infection has long been considered as one of the top three AIDS-defining opportunistic infections, alongside tuberculosis and cryptococcosis [9, 10].

In recent years, improved treatment of HIV infection with highly active antiretroviral therapy and control of the HIV/AIDS epidemic with other measures have led to a change in the epidemiology of *T. marneffei* infection, with an increasing number and proportion of cases being reported in non-HIV-infected patients who had other immunocompromising conditions [11–14]. For example, we have described the first case of fatal *T. marneffei* infection in a patient with underlying autoimmune hepatitis a few years ago [11]. However, making a diagnosis of *T. marneffei* infection requires a high index of suspicion, ordering the appropriate laboratory tests and alerting the clinical microbiology laboratory on such a suspicion, which is sometimes not easy particularly in geographical regions that are not endemic with this fungus. This is because isolating *T. marneffei* from clinical specimens often takes at least five days and most cultures will be discarded by that time.

In the last few years, next-generation sequencing (NGS) has emerged as a technology for laboratory diagnosis of many culture-negative infections [15, 16]. We have recently reported its application in confirming the first case of listeria meningitis in a patient with autoantibody against interferon gamma and fatal *Nocardia kroppenstedtii* bacteremic pneumonia and empyema thoracis as well as understanding the spectrum of Q fever, Whipple disease, fungal infections and culture-negative meningitis and encephalitis [17–21]. In this study, we describe the use of metagenomic NGS (mNGS) for rapid diagnosis of *T. marneffei* infection in a renal transplant recipient. The epidemiology of this clinical entity as well as the usefulness of mNGS for its rapid diagnosis in comparison to traditional fungal culture are also analyzed and discussed.

## Materials and Methods

### Ethical Statement

This study was approved by the Institutional Review Board of The University of Hong Kong—Shenzhen Hospital ([2022]120), and informed consent was signed.

### Index Patient

The index patient in this study was hospitalized in The University of Hong Kong—Shenzhen Hospital, Shenzhen, China. This 1400-bed multi-specialty hospital was established in 2012 and provides primary to tertiary medical services to the residents of Shenzhen city in both inpatient and outpatient settings. Supported through the policy from the government of Shenzhen, the hospital is established as a reform model medical institution in China, and many new medical technologies can be introduced to the hospital first.

### Microbiological Tests

Clinical specimens collected from the index patient were handled according to the standard operating protocols [22]. For fungal culture, respiratory specimens, including bronchoalveolar lavage fluid (BAL), tracheal aspirate and sputum, as well as abscess aspirate were each inoculated onto two Sabouraud dextrose agar (SDA) plates and one triphenyltetrazolium chloride (TTC) SDA plate. One of the SDA plates and the TTC SDA plate were incubated at 37 °C, and the other SDA plate incubated at 25 °C. The agar plates were examined daily for the first 5 days, and then twice weekly afterwards. All suspected colonies were identified based on morphological characteristics, conventional biochemical methods and matrix-assisted laser desorption/ionization-time of flight mass spectrometry (MALDI-TOF MS) Microflex LT/SH (Bruker Daltonics, Bremen, Germany) and the spectra analyzed with IVD MALDI Biotyper 4.2.100.19 and reference library DB-11897_4274 (Bruker Daltonics) [23, 24].

### Next-Generation Sequencing

BAL collected from the index patient was sent to GensKey Laboratory (Beijing, China) for NGS analysis. Interpretation of the sequencing results and determination on whether the sequences were from pathogens, latent infections or contaminants were performed by correlating them with the clinical details, laboratory data and radiological findings of the patient.

### Epidemiology of *T. marneffei* Infections in Renal Transplant Recipients

“*Talaromyces marneffei*” and “renal transplant” were used as keywords for PubMed search on 30th November, 2023. All the 20 articles were retrieved and the clinical and laboratory data of the 22 cases of talaromycosis in renal transplant recipients described in these 20 articles were analyzed [25–44].

### Statistical Analysis

The time between collection of specimen and diagnosis using NGS and culture were compared using Mann–Whitney U test.

## Results

### Index Patient

**Fig. 1:**
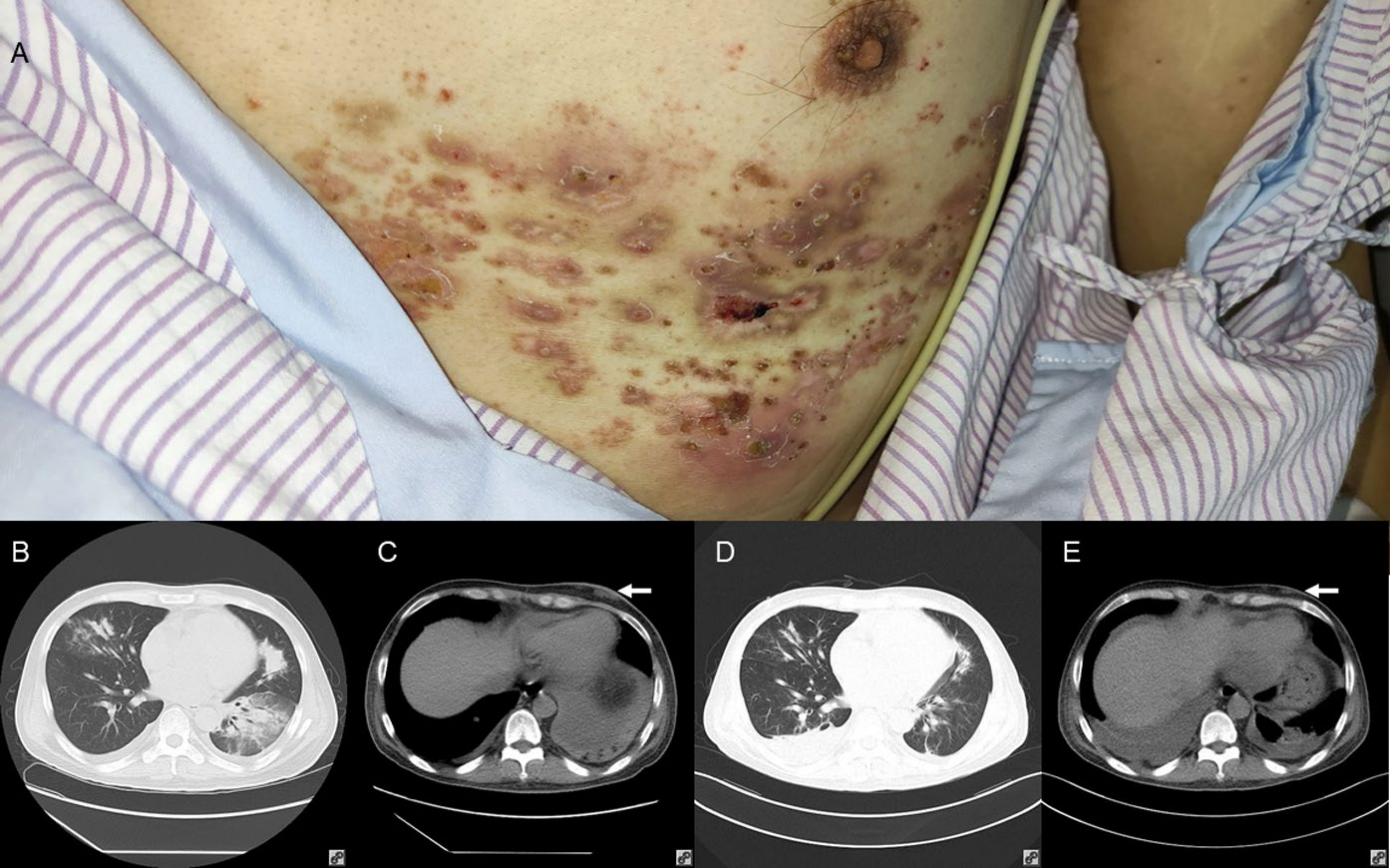
Clinical and radiological assessment of the index patient. Panel **A**: Clinical photo on admission, showing subcutaneous nodules on left chest wall and crusting due to healing herpes zoster. Panel **B**: Thoracic computerized tomography (CT) on admission, showing multiple patchy infiltrates and consolidation with air bronchograms in both lungs and bilateral pleural effusion. Panel **C**: Mediastinum window of thoracic CT on admission, showing multiple subcutaneous nodules of low density in the left chest wall (arrow). Panel **D**: Thoracic CT two weeks after commencement of antifungal therapy, showing resolving lung infiltrates and consolidation, and residual pleural effusion. Panel **E**: Mediastinum window of thoracic CT two weeks after commencement of antifungal therapy, showing shrinking of the subcutaneous nodules in left chest wall (arrow)

A 37-year-old Chinese man was admitted because of fever, cough and shortness of breath for one day. Three years ago, the patient underwent cadaveric renal transplantation because of chronic glomerulonephritis. Since then, he received tacrolimus 0.5 mg twice daily, mycophenolate 540 mg twice daily and prednisone 10 mg once daily as anti-rejection therapy. Six months ago, he had SARS coronavirus-2 infection, developed acute renal failure and was put on hemodialysis for two months. Two weeks ago, he had herpes zoster of the left T5-T7 regions and was put on valacyclovir. One day ago, he developed gradual onset of fever, cough and shortness of breath. His body temperature was 42 °C. The heart rate was 112/min and respiratory rate 17/min. Crusting due to herpes zoster as well as painless subcutaneous nodules was observed on the left chest wall (Fig. 1A). His SaO_2_ was 97.6% on room air. The total white cell count was 2.09 × 10^9^/L, with a neutrophil count of 1.68 × 10^9^/L, a lymphocyte count of 0.26 × 10^9^/L and a monocyte count of 0.13 × 10^9^/L. The patient’s hemoglobin level was 60 g/L and his platelet count was 87 × 10^9^/L. The serum creatinine level was 606 μmol/L. Liver enzymes and total bilirubin levels were normal. C-reactive protein was 133.37 mg/L, and procalcitonin was 7.42 ng/mL. Computed tomography (CT) scan of the thorax revealed multiple patchy infiltrates and consolidation with air bronchograms in both lungs, bilateral pleural effusion and multiple subcutaneous nodular foci of low density in the left thoracic wall (Fig. 1B and C). Blood culture was performed. Blood was also collected for serum galactomannan antigen and 1,3-β-D-glucan tests. Bronchoscopy was also performed and BAL was collected for bacterial, fungal and mycobacterial culture as well as mNGS. Empirical intravenous piperacillin-tazobactam, doxycycline, cotrimoxazole, voriconazole, micafungin, valacyclovir and immunoglobulin were commenced. Tacrolimus and mycophenolate were stopped and prednisone was switched to methylprednisolone, 40 mg once daily. Hemodialysis was also commenced.

After 10 h of incubation, blood culture was positive for *Acinetobacter pittii*. The optical density index of serum galactomannan was 0.86 (normal range: < 0.5). Serum 1,3-β-D-glucan was < 37.5 pg/mL. BAL for mNGS was positive for sequences of *A. pittii*, *T. marneffei*, and other oropharyngeal microbes and latent herpesviruses (Table 2). *A. pittii*, *Staphylococcus aureus*, *Streptococcus mitis* and *Streptococcus milleri* were recovered from BAL. Since BAL was positive for *T. marneffei* sequences, prolonged incubation of the BAL-inoculated plates was performed. The antimicrobial regimen was modified to intravenous meropenem for *A. pittii* bacteremia, intravenous voriconazole for *T. marneffei* infection and oral cotrimoxazole for *Pneumocystis jirovecii* prophylaxis. Fever and shortness of breath gradually subsided. After a total of six days of incubation, *T. marneffei* with characteristic thermal dimorphism and diffusible red pigment production by the mycelial form was isolated from the BAL. CT scan of the thorax performed two weeks after commencement of voriconazole showed that the lung infiltrates and consolidation were resolved and the subcutaneous nodules have also shrunk significantly (Fig. 1D and E). The patient was discharged with oral voriconazole and cotrimoxazole. There was no relapse of the illness up to the time of writing, six months after discharge.

### Epidemiology of *T. marneffei* Infections in Renal Transplant Recipients

**Fig. 2:**
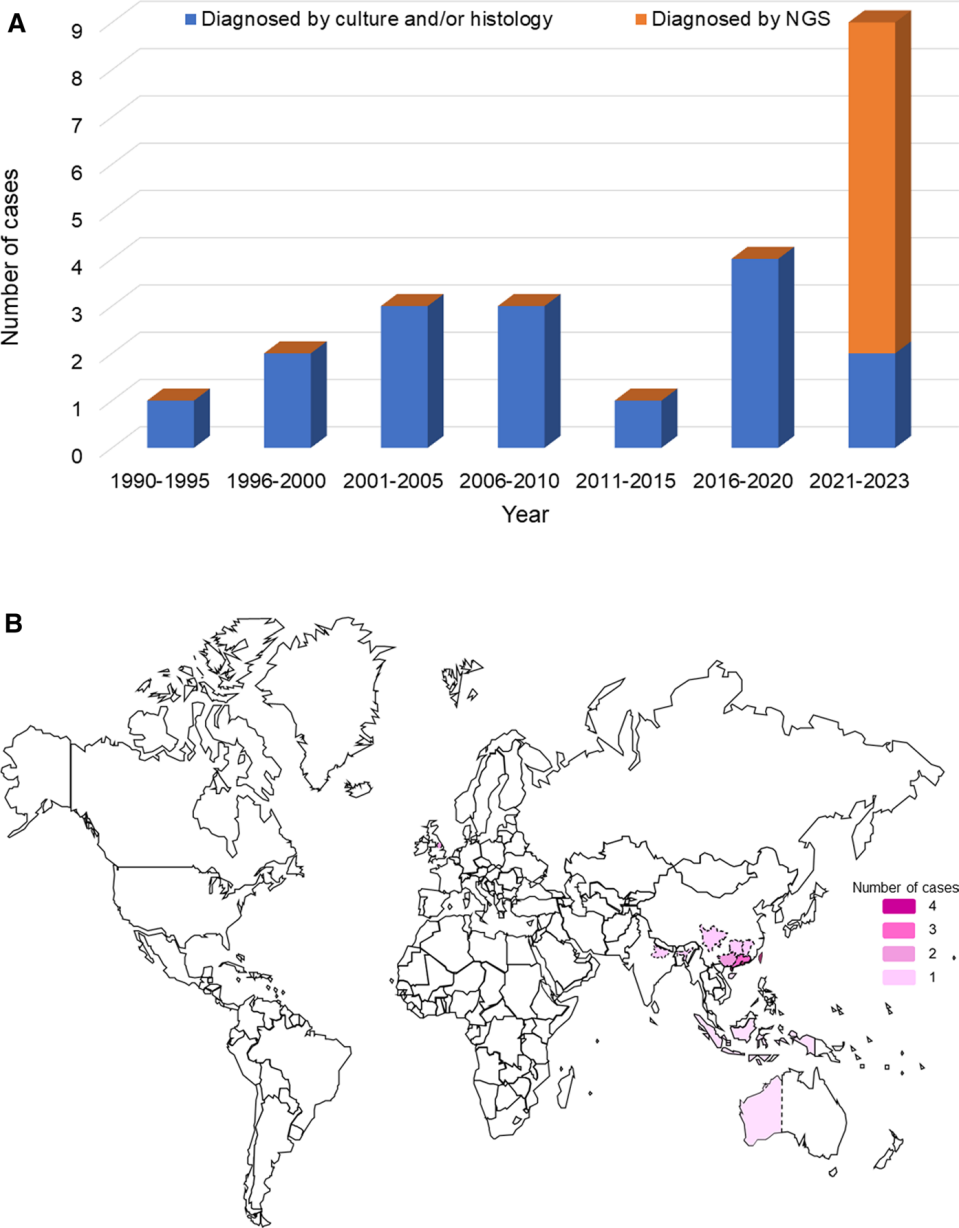
Temporal and geographical distribution of *T. marneffei* infections in renal transplant recipients. Panel **A**: Number of reported cases of *T. marneffei* infections in renal transplant recipients from 1990 to 2023. Panel **B**: Global distribution of reported cases of *T. marneffei* infections in renal transplant recipients. The number of patients is depicted in different grades of purple color

Including the index case of the present report, a total of 23 cases of *T. marneffei* infections in renal transplant recipients were identified in the literature (Table 1, Fig. 2A). These 23 cases were from the southern part of mainland China (*n* = 9), Taiwan (*n* = 4), Hong Kong (*n* = 4), India (*n* = 2), the United Kingdom (*n* = 2), Indonesia (*n* = 1) and Australia (*n* = 1) (Fig. 2B). Clinical details were available for 21 cases (Table 1). The male-to-female ratio is 15:6, and the median age is 47 (range 27–67) years. Thirteen (61.9%) of the 21 cases had disseminated *T. marneffei* infection, with pneumonia present in nine (42.9%) patients (Table 1).

**Table 1:** Clinical and laboratory characteristics of renal transplant recipients with *Talaromyces marneffei* infection

Patient number	Year	Sex/Age	Geographical region	Clinical syndrome(s) of *T. marneffei* infection	Diagnostic methods for detection of *T. marneffei*	Interval between specimen collection and diagnosis of *T. marneffei* infection (days)		Antifungal therapy	Outcome
						NGS	Culture		
1 [25]	1990	F/27	Hong Kong	Disseminated *T. marneffei* infection	Blood, urine, sputum, and marrow for culture	N/A	Not mentioned	Amphotericin B, 5-fluorocytosine	Improved
2 [26]	1998	M/43	Taiwan	Disseminated *T. marneffei* infection	Ascites and blood for culture	N/A	Not mentioned	Amphotericin B	Succumbed
3 [27]	1999	M/33	Taiwan	Disseminated *T. marneffei* infection complicated with intestinal mycoses, septic shock	Blood for culture	N/A	8	None	Succumbed
4 [28]	2003	M/47	Taiwan	Disseminated *T. marneffei* infection complicated with skin abscess, osteomyelitis	Blood and debrided specimens for culture	N/A	Not mentioned	Amphotericin B, itraconazole	Improved
5 [29]	2004	M/38	Hong Kong	Disseminated *T. marneffei* infection complicated with pneumonia, lymphadenitis	Blood and bone marrow for culture	N/A	15	Amphotericin B, itraconazole	Improved
6 [30]	2004	M/46	Indonesia	Disseminated *T. marneffei* infection	Skin lesion for culture	N/A	Not mentioned	Not mentioned	Succumbed
7 [31]	2008	Not mentioned	Hong Kong	Not mentioned	Not mentioned	N/A	Not mentioned	Not mentioned	Not mentioned
8 [31]	2008	Not mentioned	Hong Kong	Not mentioned	Not mentioned	N/A	Not mentioned	Not mentioned	Not mentioned
9 [32]	2010	F/42	Taiwan	*T. marneffei* osteomyelitis	Bone tissue for culture	N/A	Not mentioned	Amphotericin B, itraconazole	Improved
10 [33]	2011	M/67	Western Australia	Disseminated *T. marneffei* infection complicated with peritonitis secondary to perforated sigmoid colon diverticulum	Blood and peritoneal fluid for culture	N/A	3	Amphotericin B, itraconazole	Improved
11 [34]	2017	M/51	Guangdong	Disseminated *T. marneffei* infection	Blood for culture	N/A	3	Amphotericin B, itraconazole	Improved
12 [35]	2018	F/53	Greater Manchester	Lung mass due to *T. marneffei*	Lung mass tissue for culture	N/A	Not mentioned	Amphotericin B, itraconazole	Improved
13 [36]	2020	F/53	Greater Manchester	Lung mass and lymphadenitis due to *T. marneffei*	Lung mass and lymph node biopsy for culture	N/A	Not mentioned	Amphotericin B, itraconazole	Improved
14 [37]	2020	M/56	Assam	Lung mass due to *T. marneffei*	Lung mass tissue for culture	N/A	7	Amphotericin B, itraconazole	Improved
15 [38]	2021	M/34	Sichuan	Disseminated *T. marneffei* infection complicated with pneumonia	BAL for NGS and culture, blood for culture	3	Not mentioned	Posaconazole	Improved
16 [39]	2021	F/47	Hunan	Disseminated *T. marneffei* infection complicated with urinary tract infection	Blood and urine for culture	N/A	6	Voriconazole, itraconazole	Improved
17 [40]	2022	M/51	Jiangxi	*T. marneffei* pneumonia	BAL for NGS and culture	2	7	Voriconazole	Improved
18 [41]	2022	M/54	Guangdong	*T. marneffei* laryngitis	BAL for NGS and culture, sputum for culture	1	7	Voriconazole	Improved
19 [42]	2022	F/41	Bihar	*T. marneffei* skin abscess and skull osteomyelitis	Scalp lesion aspiration for culture	N/A	6	Itraconazole	Improved
20 [43]	2022	M/61	Guangxi	*T. marneffei* pneumonia	BAL for NGS	Not mentioned	N/A	Voriconazole, caspofungin	Succumbed
21 [43]	2022	M/55	Guangxi	Disseminated *T. marneffei* infection	Blood for NGS	1	N/A	Voriconazole, caspofungin	Succumbed
22 [44]	2023	M/31	Hainan	Disseminated *T. marneffei* infection complicated with pneumonia, lymphadenitis	Blood and BAL for NGS, blood for culture	1	7	Voriconazole, amphotericin B	Improved
23 [Present case]	2023	M/37	Guangdong	Disseminated *T. marneffei* infection and pneumonia complicated with septic shock	BAL for NGS and culture	1	6	Voriconazole, micafungin, valacyclovir	Improved

*F* female, *M* male, *N/A* not applicable, *NGS* next-generation sequencing

### Rapid Diagnosis of *T. marneffei* Infections in Renal Transplant Recipients by NGS

**Fig. 3:**
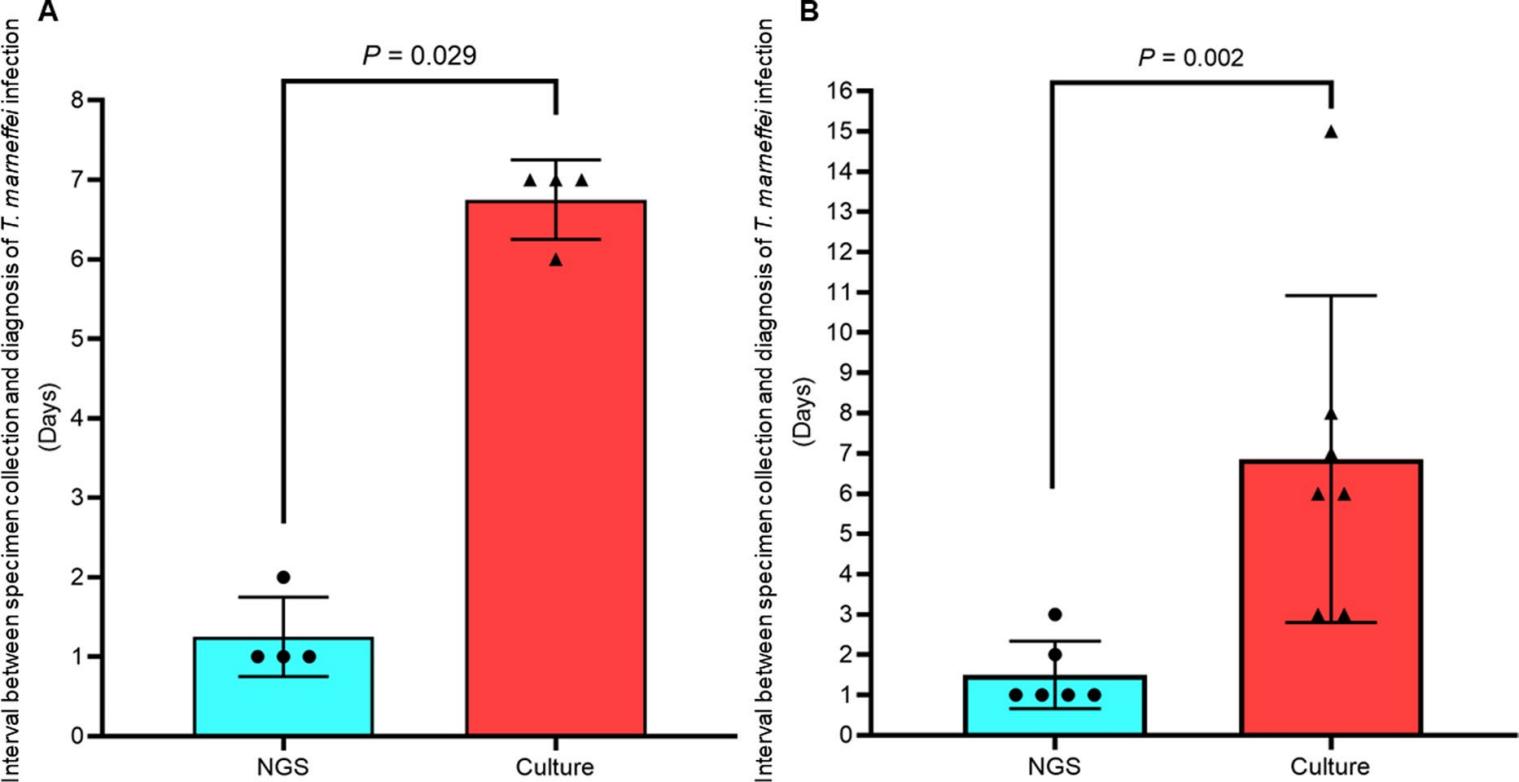
Comparison of time taken for diagnosis of *T. marneffei* infection by mNGS and culture. Panel **A**: The 4 cases diagnosed by both mNGS and culture. Panel **B**: The 6 cases diagnosed by mNGS and the 7 cases diagnosed only by culture

Among all the cases of *T. marneffei* infections in renal transplant recipients, seven were diagnosed by mNGS (Cases 15, 17, 18, 20, 21, 22 and 23, Table 1), with a median time between collection of specimen and diagnosis of 1 (range 1–2) day. In five of the seven cases, *T. marneffei* was also recovered from specimens sent for culture. The time between collection of specimen and positive culture result was mentioned in 4 cases, the median of which was 7 (range 6–7) days, which is significantly longer than that by mNGS (*P* = 0.029) (Fig. 3A). As for the other cases of *T. marneffei* infections in renal transplant recipients diagnosed by culture in the literature, the time between collection of specimen and positive culture result was mentioned in 7 cases, the median of which was 6 (range 3–15 days, which is also significantly longer than the cases diagnosed by mNGS (*P* = 0.002) (Fig. 3B).

## Discussion

In this study, we describe an emergence of *T. marneffei* infections in renal transplant recipients and showed the role of mNGS in picking up difficult-to-diagnose cases. Among the 23 cases of *T. marneffei* infections in renal transplant recipients, 20 were from *T. marneffei* endemic areas (Fig. 2B). The nine patients from mainland China were from Guangdong (*n* = 3) (Cases 11, 18, and 23), Guangxi (*n* = 2) (Cases 20 and 21), Hainan (*n* = 1) (Case 22), Hunan (*n* = 1) (Case 16), Jiangxi (*n* = 1) (Case 17) and Sichuan (*n* = 1) (Case 15), which were all located in the southern part of China (Fig. 2B). The two patients from India were from Dibrugarh, Assam (Case 14) and Patna, Bihar (Case 19) respectively. These two regions are in northeastern India (Fig. 2B), where *T. marneffei* is endemic. For the three patients that were from non-*T. marneffei*-endemic areas, two were from the United Kingdom (Cases 12 and 13) and one from Australia (Case 10). It is notable that although the two patients from the United Kingdom resided in Manchester, they had travel histories to China. One had traveled to South China whereas the other to Beijing, Xi’an, Sichuan and Yunnan. As for the Australian from Perth, he also had travel histories to Hanoi, HaLong Bay and Sapa of Vietnam, where *T. marneffei* is endemic. It is interesting to note that the number of cases of *T. marneffei* infections in renal transplant recipients ranged from one to four cases per five years from 1990 to 2020, but increased rapidly in the last few years, with already nine cases reported from 2021 to 2023 (Fig. 2A). Seven of these nine cases were diagnosed by mNGS, indicating that this technology has resulted in an apparent emergence of this disease entity and that this infection has probably been underestimated previously. It is also important to note that among these seven cases, *T. marneffei* sequence reads were positive in the BAL and blood samples in six (Cases 15, 17, 18, 20, 22 and 23) and two (Cases 21 and 22) patients respectively. Although formal comparison between the sensitivities of different samples is yet to be performed, it seems that BAL is a reasonably promising specimen when the patient had pneumonia.

In addition to picking up more cases of *T. marneffei* infections in renal transplant recipients, NGS is also crucial in providing a rapid diagnosis for this clinical entity. Definitive laboratory diagnosis of *T. marneffei* infection was traditionally achieved by isolating the fungus in clinical specimens and recognizing its unique phenotypic characteristics of thermal dimorphism, diffusible red pigment production by its filamentous form as well as microscopic features of the yeast and filamentous forms, etc. [23]. Recently, we have demonstrated that MALDI-TOF MS is useful for the identification of *T. marneffei* cultures as [3, 24]. However, isolating the fungus from clinical samples usually take at least five days. For example, for the patients with *T. marneffei* infections in renal transplant recipients described and reviewed in the present study, a median of six or seven days was required (Fig. 3). On the other hand, when mNGS was used, the laboratory diagnosis was made within one day in most of the cases (Fig. 3). In fact, for the index case described in the present study, *T. marneffei* was isolated only because prolonged incubation of the BAL sample was requested due to the fact that *T. marneffei* sequences were observed in the BAL submitted for mNGS analysis. If the sample was not sent for mNGS analysis, the sample would probably have been discarded and the diagnosis markedly delayed or missed. It is important to note that during mNGS analysis, in addition to the microbes that cause the present clinical problem, many other colonizers and/or contaminants are usually also being sequenced. For example, in the present case, in addition to *A. pittii* (also recovered in blood culture) and *T. marneffei*, sequence reads of other oropharyngeal microbes and latent herpesviruses were also detected in the patient’s BAL by mNGS analysis (Table 2). Results of mNGS analysis must be interpreted together with the clinical context of the patient in order to determine which ones are the real causative agents.

**Table 2:** Microorganisms detected in bronchoalveolar lavage fluid of the index patient by mNGS

Microorgansims	Number of reads
*Acinetobacter pittii*	1,649,958
*Streptococcus constellatus*	68,863
*Staphylococcus aureus*	61,410
*Talaromyces marneffei*	200
Varicella zoster virus	21,541
Herpes simplex virus-1	3693
Epstein-Barr virus	773
Human herpes virus-7	273
Human herpes virus-6B	12
Torque teno virus	66
Cytomegalovirus	121
*Streptococcus salivarius*	598,829
*Streptococcus oralis*	184,690
*Prevotella melaninogenica*	702,644
*Actinomyces graevenitzii*	739,902
*Peptostreptococcus stomatis*	364,570
*Neisseria elongate*	54,683
*Alloprevotella tannerae*	214,813
*Gemella morbillorum*	142,707
*Porphyromonas gingivalis*	118,646
*Solobacterium moorei*	147,988
*Candida parapsilosis*	24

A high index of suspicion is required for making an early diagnosis of *T. marneffei* infections in HIV-negative patients. The clinical presentation of *T. marneffei* infections is always non-specific, with fever, weight loss, lymphadenopathy and hepatosplenomegaly being some of the more common symptoms and signs. It makes it particularly difficult for clinicians who are not familiar with this fungal infection. For example, in the present patient, the skin lesion might have prompted some physicians to perform skin biopsy and examine it microscopically, which may reveal the characteristic yeast cells with septum formation and give a clue to the culprit [45]. In addition to the difficulty in isolating the fungus, it has been shown that *T. marneffei* infection often resulted in false-positive galactomannan antigen test [46], which will give the clinician a wrong impression that the patient may be suffering from aspergillosis, as invasive *Aspergillus* infections are as important as talaromycosis in these immunocompromised patients. For the underlying disease, traditionally this infection manifested mostly in HIV-positive patients. In recent years, as we have been using more immunosuppressive regimens, such as targeted therapy; and technology advancement has also resulted in the recognition of more primary immunodeficiency syndromes and their corresponding genetic origins, *T. marneffei* has emerged in certain groups of HIV-negative patients [11–14, 17]. As for the geographical distribution, *T. marneffei* infections occurred almost exclusively in Southeast Asia, where *T. marneffei* infection is regarded as an AIDS-defining condition in HIV-positive patients. As shown in the present study, only 3 of the 23 renal transplant recipients with *T. marneffei* infections were from non-endemic areas; but in fact, they all have travel histories to Southeast Asia [33, 35, 36]. As a result of all these factors, making a diagnosis of *T. marneffei* infection is particularly difficult in HIV-negative patients, especially if the individual is not from endemic regions. With globalization and more frequent travels, we anticipate that there will be more *T. marneffei* infections in the western world. A high index of suspicion and ordering the appropriate laboratory tests are essential for timely commencement of proper antifungal treatment for *T. marneffei*.
